# Ferrocene probe-assisted fluorescence quenching of PEI-carbon dots for NO detection and the logic gates based sensing of NO enabled by trimodal detection

**DOI:** 10.1038/s41598-024-61117-z

**Published:** 2024-05-06

**Authors:** S. Priya, Sheela Berchmans

**Affiliations:** 1 NSS College, Nemmara, Palakkad India; 2 Electrodics and Electrocatalysis Division, CSIR-Central Electrochemical Research Institute, Karaikudi, Tamilnadu 630006 India

**Keywords:** Chemistry, Materials science, Nanoscience and technology

## Abstract

Our research demonstrates the effectiveness of fluorescence quenching between polyethyleneimine functionalised carbon dots (PEI-CDs) and cyclodextrin encapsulated ferrocene for fluorogenic detection of nitric oxide (NO). We confirmed that ferrocene can be used as a NO probe by observing its ability to quench the fluorescence emitted from PEI-CDs, with NO concentrations ranging from 1 × 10^–6^ M to 5 × 10^–4^ M. The photoluminescence intensity (PL) of PEI-CDs decreased linearly, with a detection limit of 500 nM. Previous studies have shown that ferrocene is a selective probe for NO detection in biological systems by electrochemical and colorimetric methods. The addition of fluorogenic NO detection using ferrocene as a probe enables the development of a three-way sensor probe for NO. Furthermore, the triple mode NO detection (electrochemical, colorimetric, and fluorogenic) with ferrocene aids in processing sensing data in a controlled manner similar to Boolean logic operations. This work presents key findings on the mechanism of fluorescence quenching between ferrocene hyponitrite intermediate and PEI-CDs, the potential of using ferrocene for triple channel NO detection as a single molecular entity, and the application of logic gates for NO sensing.

## Introduction

Nitric oxide (NO) is an intracellular signaling molecule. Until 1987, it was considered to be a strong environmental pollutant, but it was later found to be an endothelium-derived relaxing factor^1–4^. NO has been found to play an active role in the cardiovascular system, neurotransmission, immune system, wound healing, etc^5–9^. Due to the enormous interest in the biological role of nitric oxide, there is a need for analytical techniques that enable the detection and quantification of NO^10–23^. The most common analytical approach for NO measurement includes spectroscopic and electrochemical techniques ^24,25^; both techniques enabled the direct measurement of NO. Electrochemical techniques are more suitable for in situ monitoring of NO with low sample interference and miniaturised platforms. Since the biomolecule NO is a radical species, it is highly reactive with a half-life of 10 s in physiological media. NO is detected electrochemically by electrooxidation and electroreduction. Electrochemical NO detection requires appropriate reaction time, sensitivity and a suitable detection limit. Due to its wide biologically relevant concentration range (pM–μM), selectivity is another important concern when developing a NO sensor, as a large number of electro-active species present in the physiological environment can falsely contribute to the sensor's current response Therefore, electrochemical sensors must be designed to meet these specific challenges^26–28^. Most of the approaches used for NO detection involve the amperometric method, where NO is oxidized or reduced on the electrode surface. During oxidation process, NO converted to nitrosonium ions (NO^+^), the amount of current produced corresponds to the concentration of NO ^29^.

Spectroscopic techniques include electron paramagnetic resonance spectroscopy (EPR)^30^, fluorescence^31^, GC-MS^32^, spectrophotometric detection with hemoglobin and via nitrite-azo coupling reaction^33^. The colour-based sensing approach is simple, inexpensive and the signal obtained is based on naked eye detection and can be considered for point-of-care testing (POCT). However, the detection sensitivity is low. The most common colorimetric method for detecting NO is based on the Griess assay, which indirectly measures NO as nitrite or nitrate. It is a two-step diazotization method and the product formed shows absorption at 540 nm^34^. The chemiluminescence method enables rapid, sensitive and real-time monitoring of NO detection, where NO reacts with ozone to produce excited nitrogen dioxide that spontaneously emits photons and decays back to its lower energy state. Another method of chemiluminescence involves the use of luminol as a chemiluminescent agent^35,36^. Fluorescence technique offers several advantages compared to other quantification techniques, including sensitivity, convenience, low cost etc^37,38^. Fluorescent probes can react directly and selectively. Selective and sensitive monitoring of NO using fluorescent probes plays a major role in studying the production of NO in vivo and explaining its biological significance. A sufficiently understood principle, simple operating technology and fast measurement values ​​made fluorescence technology a dominant tool for detecting biologically important gas molecules. Therefore, fluorescent nanoprobes are suitable for in vivo imaging applications ^39–41^.

To date, several NO fluorescent probes have been described^31^. These fluorescent probes are based on two different approaches: (1) uses organic fluorophores with electron-rich centers such as diaminobenzene, which reacts with an oxidized product of NO to form an electron-poor product such as triazole to modulate the emission of the fluorophore ^42–46^. (2) The second approach involves the use of transition metals such as Co (II), Fe (II), Ru (II), Cu (II) or Rh (II) ^47–57^. Although many methods have been developed to address issues such as spatiotemporal resolution, sensitivity, selectivity and biocompatibility, concerns remain regarding certain issues such as extending biocompatibility over long periods of time to improve cell viability, sensitivity and selectivity down to picomolar levels and to maintain sensing technologies that can be linked to the membrane, as NO release is regulated by the plasma membrane. When developing sensor strategies that can be connected to the membrane, sensor probes with dimensions < 10 nm such as semiconductor quantum dots^44^, silicon-dye hybrid nanoparticles^45^, metal nanoclusters^46^, carbon nanodots (CDs) and organic dye-lipid conjugates^47^ play a major role in biological NO detection. In this context, the production and characterization of CDs suitable for selective NO detection is of great importance.

Photoluminescent (PL) carbon nanodots (CDs) have recently attracted considerable attention due to their biocompatibility for biomedical applications and green synthesis^4–7^^.^ Among various techniques, microwave-assisted pyrolysis of a carbon source is a low-cost, efficient, and simple method to produce CDs^52–57^. Surface passivated CDs with diameters less than 10 nm are widely used for biomarking and sensing applications due to their stable PL, biocompatibility, broad excitation, and tunable emission wavelength^14^. Additionally, CDs are nontoxic and have been shown to retain the functional units of the carbonaceous precursors used in synthesis. In addition, they are amenable to further surface modifications that affect the recognition units that can be attached to their surface^58,59^. Microwave synthesised sulfur- and nitrogen-doped carbon dots and CDs synthesised from citric acid and ethylenediamine have been reported as NO sensors^60,61^. The ratiometric sensing of NO has been reported by constructing a FRET system using CDs with carboxylic acid functionalities further modified with a naphthalimide and o-phenylenediamine recognition element^62^. The methods described have problems such as low sensitivity, long reaction times or complex synthesis procedures. The aim of this work is to synthesis CDs with sizes < 10 nm suitable for selective NO detection in the presence of the molecular probe ferrocene, which we have previously used in our laboratory for bimodal NO detection using spectrophotometric and electrochemical approaches^63–66^. Quenching the fluorescence of CDs by NO in the presence of ferrocene has added another mode for the selective detection of NO. In addition, we were able to provide insight into the mechanism of fluorescence-based detection of NO.

In this study, we integrated CDs together with a NO sensing element, β-hydroxypropylcyclodextrin encapsulated ferrocene (Fc_aq_) to fabricate a nanosensor for NO. The selective response of Fc_aq_ to NO and other ions makes it suitable for practical applications. The mechanism of fluorescence quenching of PEI-CDs during ferrocene-mediated reduction of NO is described and attributed to the ferrocene hyponitrite intermediate^67^ formed during the reaction, which is similar to the observed phenomenon of quenching of photoexcited CDs by the reaction intermediate. This involves a photoinduced electron transfer (PET)^68^ from CDs to the intermediate product. This work has added another modality for the detection of NO using ferrocene as a probe, in addition to the electrochemical and colorimetric approaches previously reported by our laboratory ^63–66^. Multimodal detection of an analyte provides complementary information and increases the robustness and accuracy of the analysis. In addition, the multimodal detection capability improves the efficiency of analysis with minimised assumptions, meets more analytical requirements, and provides improved flexibility for applications. The feasibility of triple-modal detection can potentially meet more analytical needs. The colorimetric method can be a quick qualitative test. Based on this quantitative analysis, either fluorescence-based or electrochemical approaches can be performed. Efficiently molecular logic gates are chemical units that can mimic the function of the basic operations of an electronic computer. Sensor output can be performed analogously to Boolean logic operations by intelligently arranging the molecular entities and their transduction strategies. Sensing could be done in a logically controlled manner through the combination of biomolecules and logic gates. Interestingly, it turns out that three-channel detection of NO is analogous to the operation of three different logic gates.

## Experimental

### Materials

Linear polyethyleneimine (PEI) (MW25,0000), citric acid and β-hydroxy propyl cyclodextrin were purchased from Sigma-Aldrich. Sodium nitrite and Ferrocene were purchased from Alfa Aesar. All other reagents were of analytical grade and used as received. All experiments were carried out using Millipore water.

### Synthesis of PEI-CDs, cyclodextrin encapsulated ferrocene (Fc_aq_) and nitric oxide (NO)

Highly photoluminescent PEI-CDs were prepared using the green route of microwave assisted pyrolysis. Briefly, 1 g of citric acid was first dissolved in 10 mL of water; 0.5 g of linear PEI was added and stirred for 15 min to obtain a clear, homogeneous solution. Then the beaker containing this solution is placed in the rotating platform of the microwave oven and heated at 180 ^○^C for 6 min. The resulting yellow-orange solution was then diluted and dialysed. This solution was lyophilised into powder for further analysis. The preparation time was optimised based on the intensity of emission of prepared PEI-CDs at different time intervals. Maximum intensity was observed for PEI-CDs prepared by 6 min of irradiation.

Ferrocene was dissolved in water by mixing β-hydroxypropyl cyclodextrin (Cd) and ferrocene in a molar ratio of 1:1. Ferrocene dissolved in the presence of Cd is referred to as Fc_aq_ in this work. It is known that sodium nitrite (NaNO_2_) spontaneously undergoes a disproportionation reaction under acidic conditions (pH < 4) and produces free NO. Therefore, we chose NaNO_2_ as NO precursor^69^ and also, conducted experiments in which we prepared a standard solution of NO by purging NO gas in degassed water for 1 h. All experiments were carried out in deaerated conditions. All electrochemical studies were carried out using glassy carbon as the working electrode, which was modified by PEI-CDs by drop casting and subsequent drying.

Fluorescent quenching studies of PEI-CDs were performed in 96 well multi-plate reader and 50 μL of PEI-CDs of concentration 0.5 mg/mL were taken in different wells and 50 μL of prepared Fc_aq_ was added to each well. The pH of the solution is adjusted to 3 and the fluorescent spectra are recorded by varying the concentrations of NO.

### Instrumentation

FT-IR spectra of the samples were recorded using a Tensor FT-IR spectrophotometer from Bruker Optics. Spectra were recorded in the wave number range from 400 to 4000 cm^−1^. UV–Visible spectra were recorded at room temperature in the wavelength range 250–800 nm using the Cary 5000 UV–Vis-NIR spectrophotometer (Varian). The spectra were recorded using thoroughly cleaned quartz cuvettes. Fluorescence was recorded using the TESCAN infinite M200PRO multiplate reader. Cyclic voltammograms were recorded with PG129 STAT 12/30/302,Autolabpotentiostat/galvanostat, using GPES software version 4.9.007.The size and morphology of the prepared PEI-CDs were studied using Transmission electron microscopy (TEM) using Technai 20G2. PEI-CDs to be analysed is drop casted on carbon coated copper grid. After drop casting it is dried under infrared lamp.

## Results and discussions

### Characterisation of PEI-CDs

In this work, we synthesised PEI-CDs under different microwave irradiation times based on previous reports^47,51^. PEI-CDs were prepared by mixing 1 g of citric acid and 0.5 g of linear PEI at different irradiation times, namely 3 min, 6 min, and 10 min. Thus, prepared PEI-CDs exhibit excellent water soluble and photoluminescent properties. The intensity of emission was found to be high for PEI-CDs prepared with a microwave irradiation time of 6 min. PEI-CDs prepared with irradiation times of 3 and 10 min show weak photoluminescence, which is due to incomplete surface passivation before 6 min and large particle formation after 6 min^47^. Although the origin of photoluminescence from PEI-CDs is not clearly understood, there is growing evidence that the emission arises from radioactive recombination of excitons located at the surface energy traps^70^. The influence of pH of PEI-CDs was also tested. PL intensity was found to increase with decreasing pH (weakly acidic media) and exhibit weak fluorescence activities at other pH values. The PL intensity was found to be high in the pH range of 3–5. Therefore, the pH value for the sensor medium was set to 3, since at this pH value the maximum emission was observed upon excitation at 360 nm. It was found that the emission intensity decreases below pH 3 because too many positive charges on the PEI-CDs surface could inhibit the excited states of PEI-CDs.

### Electron transfer properties of PEI-CDs

**Figure 1:**
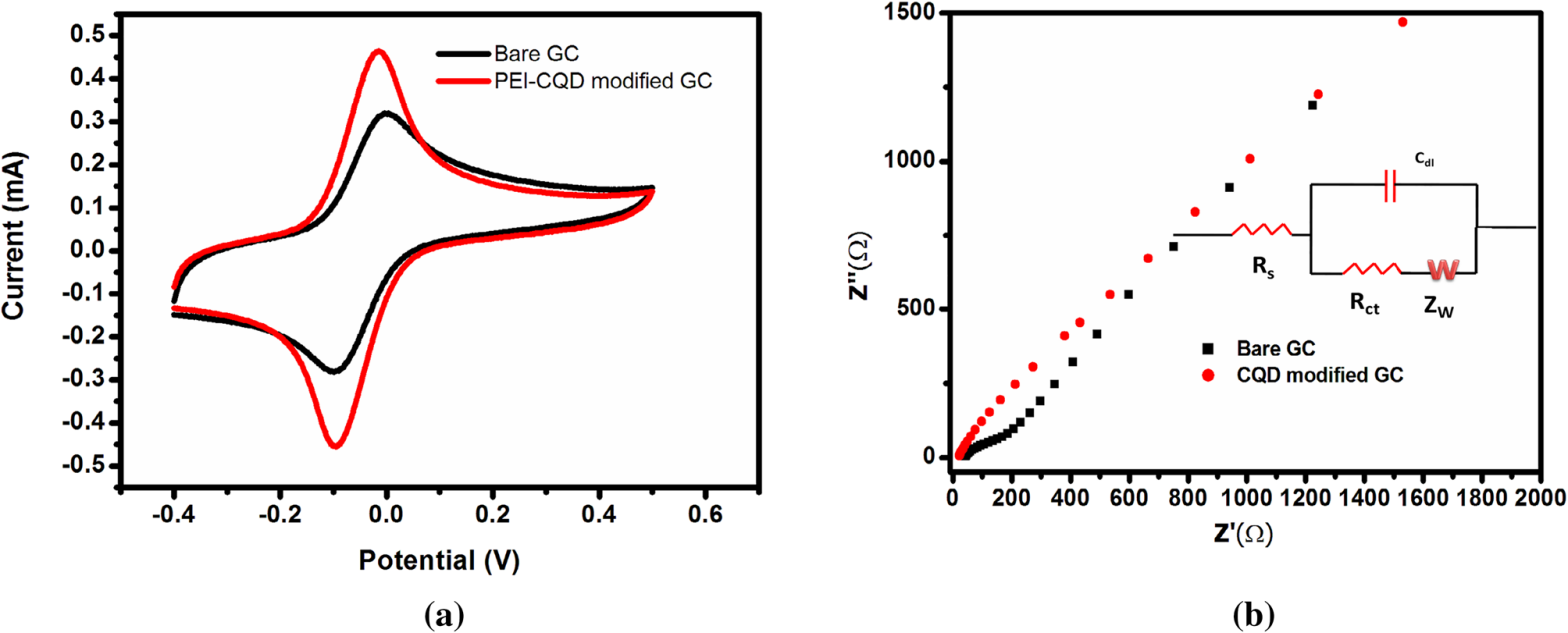
(**a**) Cyclic voltammogram and (**b**) impedance plots-Nyquist plots in equal concentrations (0.5 mM) of potassium ferrocyanide and potassium ferricyanide aqueous solution as redox probe in pH  3 for bare GCE (black) and PEI-CDs modified GCE (red), inset shows the corresponding equivalent circuit.

Figure 1a shows the cyclic voltammetric response of bare and PEI-CDs modified glassy carbon electrodes (GCE) in the presence of Fe(CN)_6_^3^^−^^/4^^−^ as a redox probe at pH 3 condition. The aim is to study the electrochemical behaviour of PEI-CDs under the same pH conditions in which we conduct fluorescence studies. Cyclic voltammograms of bare GCE show a reversible wave which is the typical response of GCE in the presence of the redox couple with ΔEp = 89 mV. After PEI-CDs modification, the current response was found to increase by 1.4fold and ΔEp decreased to 72 mV, showing an improvement in electron transfer kinetics compared to bare GCE. This observation was further confirmed by impedance spectroscopy. The electron transfer ability of the modified layer can be analysed by measuring the charge transfer resistance. From Fig. 1b it is clear that bare GCE shows a linear behaviour in the low-frequency region and small semicircle behaviour in the high frequency region with an Rct value of 89 Ωcm^−^^2^ indicating that the electron transfer process is reversible and diffusion controlled. However, after PEI-CDs modification, the Rct value was found to be greatly reduced, indicating a decrease in charge transfer resistance due to the higher conductivity of PEI-CDs^71^. The inset of the figure shows the equivalent circuit with Rs—solution resistance, Rct—charge transfer resistance, Cdl—capacitance and Zw—Warburg impedance. The active surface area of the electrode calculated using Nicholson Shain equation was found to be 1.9 × 10^–3^ cm^2^ for the modified electrode and1.4 × 10^–4^ cm^2^ for the unmodified electrode (see SI for calculation)**.**

### Surface characterization of PEI-CDs

**Figure 2:**
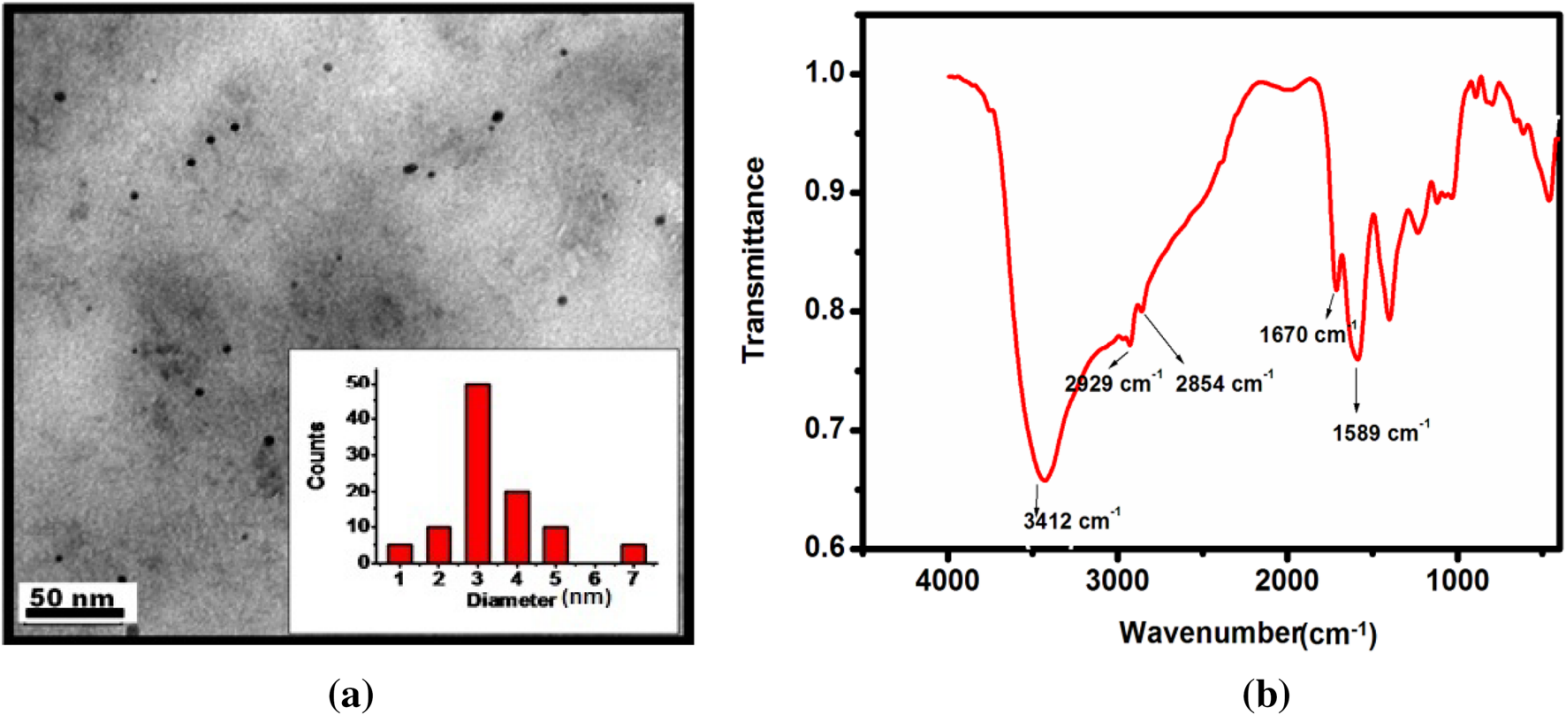
(**a**) TEM image of PEI-CDs, (**b**) IR of PEI -CDs.

The TEM image showed in Fig. 2a shows that the formed PEI-CDs have a size distribution between 3 and 7 nm (inset of Fig. 2a). FT-IR spectra were recorded to identify the functional groups on the PEI-CDs surface. Polyethyleneimine usually have a C–N stretching at 1136 cm^−1^, an NH-bending at 1500 cm^−1^ from primary amine.CH stretching and bending vibration modes are observed between 2850 and 2900 cm^−1^. But after surface functionalisation with CDs, the formation of the amide bond results in a new peak at 1670 cm^−1^ ^72^. As shown in Fig. 2b, the new peak at 1670 cm^−1^ corresponds to the C=O stretching frequency of the amide group, as well as the peaks at 1589 cm^−1^ and a broad band at 3412 cm^−1^ indicate the existence of the N–H group. The band at 2854 cm^−1^ corresponds to the C–H stretching and deformation bands. During the pyrolysis process, the carboxylic group of citric acid transformed into the amide group, which resulted in the chemical grafting of PEI on the carbon dot surface. In this way, grafted PEI helps the carbon dots become water soluble and also passivates the surface of the carbon dots to enhance photoluminescence.

Zeta potential analysis of the prepared PEI-CDs at pH 3 shows that PEI-CDs have positive charge distribution on their surface (see Supplementary Fig. S1). This positive surface charge barrier on PEI-CDs prevents positively charged quenchers from approaching the PEI-CDs. PEI-CDs show a quantum yield of 18% when excited at 360 nm compared to reference quinine sulphate. The fluorescence of PEI-CDs shows linearity in the range 0.1–0.5 mg/mL, after which it shows deviation (see Supplementary Fig. S2). Therefore, 0.5 mg/mL was chosen as the optimal concentration of PEI-CDs for NO detection.

### Fluorescence response of PEI-CDs in the presence of nitric oxide and sensing mechanism via quenching

**Figure 3:**
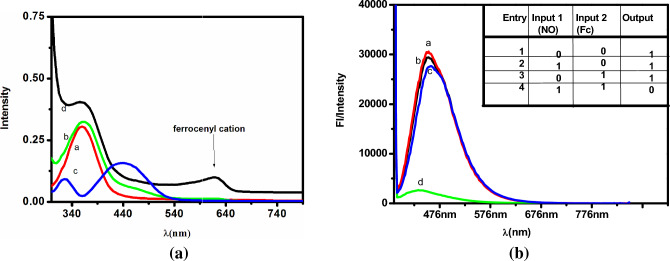
(**a**) UV–Vis absorption spectra (a) PEI-CDs, (b) Fc_aq_, (c)PEI-CDs + Fc_aq_, (d) PEI-CDs + Fc_aq_ + NO. (**b**) Fluorescence spectra (λ_excitation_ = 360 nm) (a) PEI-CDs, (b) PEI-CDs + Fc_aq_, (c) PEI- CDs + NO, (d) PEI- CDs + Fc_aq_ + NO.

Fluorescence quenching of PEI-CDs by NO is enabled in the presence of Fc_aq._ When NO reacts with Fc_aq_, a blue coloured intermediate is formed, this leads to the quenching of the fluorescence of PEI-CDs. The fluorescent probe shows absorption at 360 nm and emission at 420 nm, which is in the visible region and is valuable for biological applications (see Supplementary Figs. S3 and S4). Figure 3a shows the UV–Vis spectra of PEI-CDs, Fc_aq_, PEI-CDs + Fc_aq_ and PEI-CDs + Fc_aq_ + NO (the blue coloured intermediate formed between Fc and NO). From the UV–Vis spectra, it is clear that the absorption intensity and wavelength of PEI-CDs at 360 nm do not shift with the addition of Fc_aq_. Figure 3b shows the emission spectra of PEI-CDs in the presence of Fc_aq_, NO and Fc_aq_ + NO, indicating that the emission at 420 nm was not affected by the individual addition of Fc_aq_ or NO but was quenched by the formation of the blue coloured intermediate ferrocene hyponitrite was present when Fc_aq_ and NO were present together. NO reacts with Fc_aq_ to form a blue colour intermediate, based on which we previously reported a colorimetric sensor for NO^56^. This blue-coloured intermediate ferrocene hyponitrite leads to a reduction in the emission intensity of PEI-CDs. This suggests the possibility of establishing a quantitative connection with the increased quenching behaviour of Fc_aq_ + NO with increasing NO concentrations, under a constant ferrocene concentration. Therefore, a combination of PEI-CDs and Fc_aq_ at pH 3 is chosen for the fluorogenic detection of NO.

**Figure 4:**
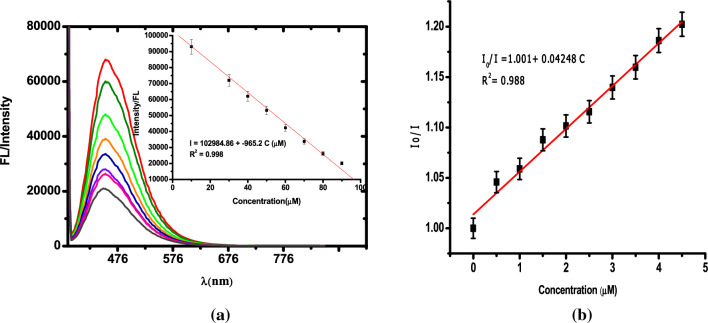
(**a**) Fluorescence spectra upon addition of 10 μM NO at pH = 3 (PEI-CDs-0.5mg/mL). Inset shows the calibration plot. (λ_exc_ = 360nm). (**b**) Stern–Volmer plot—each addition corresponds to 500 nM.

**Figure 5:**
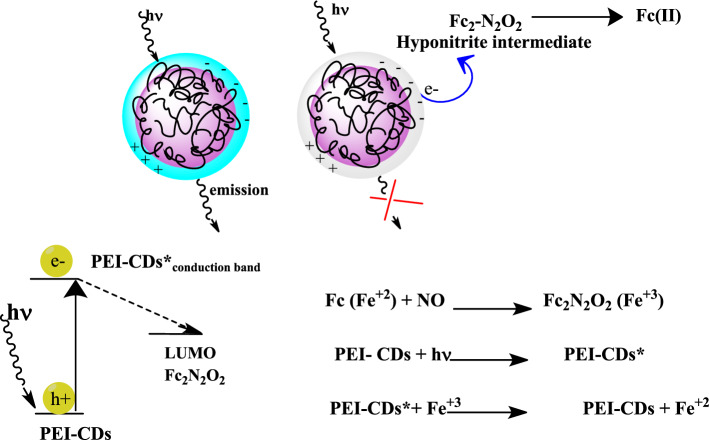
Fluorescence quenching of PEI-CDS in the presence of hyponitrite anion.

The response rate of the fluorescence signal of PEI-CDs is quenched to 85% upon addition of10 μM of NO and remains stable in the following one-hour observation. This result shows that the quenching of PEI-CDs in the presence of Fc_aq_ + NO is rapid and stable. With each addition of NO, the quenching was found to be stable and the percentage deviation was less than 5%. Figure 4a shows the PL spectra of PEI-CDs + Fc_aq_ in the presence of NO. With each addition of 10 μM NO, there was a proportional increase in the degree of quenching. The inset in Fig. 4a shows the intensity vs NO concentration plot, and quenching was found to be linear for the additions. Although the absorption of Fc_aq_ (430 nm) falls within the emission range of PEI-CDs (447 nm), no effective quenching is observed during the addition of Fc_aq_ to PEI-CDs. Furthermore, there is no spectral overlap between PL of PEI-CDs and the absorbance of the quencher (blue coloured intermediate absorbs at 618 nm), so energy transfer is unlikely. The observed quenching is therefore caused by the photo-induced electron transfer from PEI-CDs to the ferrocene hyponitrite intermediate (Fig. 5)^73–77^^.^

Studies by Wang et al. showed that carbon quantum dots can act as electron acceptors or electron donors^78–80^. Their photoluminescence studies showed that both electron donor and electron acceptor can cause quenching of PEI-CDs. In the present case, PEI-CDs act as an electron donor and the formed ferrocene hyponitrite intermediate acts as an electron acceptor. This was confirmed by our electrochemical studies using bare and PEI-CDs modified GCE. The ferrocene mediated NO reduction occurs at a potential of −0.3 V vs. Hg/Hg_2_SO_4_^64,65^. The blue colour complex formed between Fc_aq_ and NO is reduced at −0.3 V, resulting in catalytic regeneration of ferrocene with release of N_2_O and an increase in reduction currents. This shows that the intermediate ferrocene hyponitrite acts as a good electron acceptor. PEI-CDs modified GCE exhibits high catalytic current response compared to bare GCE (see Supplementary Figs. S5 and S6) indicating improved catalytic performance. Since photoluminescence can be quenched by the presence of an electron donor or acceptor, the presence of a blue colour intermediate leads to the quenching of PEI-CDs in the present case. Supplementary Fig. S7 shows the absorption spectra recorded before and after photo-excitation of PEI-CDs in the presence of an intermediate. There is a decrease in the absorption intensity of the quencher (at 618 nm), showing the dissociation of the intermediate due to photo-induced electron transfer from the conduction band of PEI-CDs to the LUMO of the intermediate. This intermediate was identified as a hyponitrite complex of ferrocene^67^.

Our electrochemical studies showed that the catalytic regeneration of ferrocene occurs after the electrochemical reduction of the intermediate. Therefore, we expect the same mechanism in the photoinduced reduction of the intermediate. During photoinduced electron transfer from PEI-CDs to the hyponitrite intermediate, reduction occurs, leading to the regeneration of ferrocene. The Stern–Volmer plots (Fig. 4b) showed a linear relationship in the case of PL quenching. I_o_ and I are the PL intensities of PEI-CDs in the absence and presence of a quencher. The Stern–Volmer coefficient was K_sv_ = 4.248 × 10^−2^ M^−1^ (K_sv_ = τ_F_^o^ k_q_). From the reported work, the average decay function (τ_F_^o^) of PEI-CDs was found to be 4ns^78^. The average bimolecular rate constant k_q_ for the quenching of the luminescence emissions from PEI-CDs by the intermediate is therefore on the order of 1.06 × 10^6^ M^−1^ s^−1^.This rate constant value shows how quickly the nanodots respond to NO detection. This observed linearity in the Stern–Volmer plot suggests the use of PEI-CDs + Fc_aq_ as an excellent fluorescent probe for NO detection.

###  Interference effect

**Figure 6:**
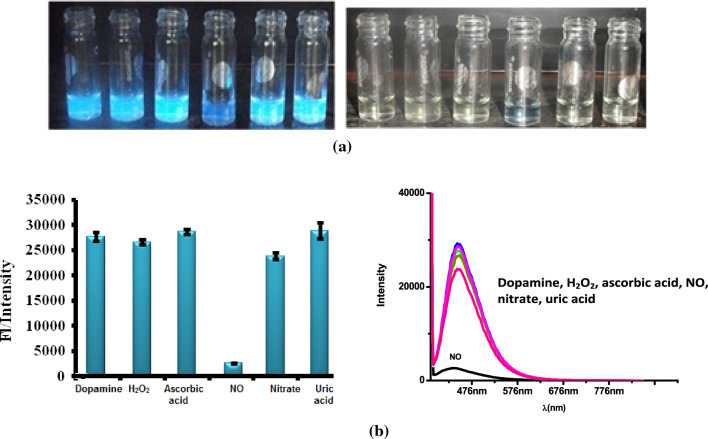
(**a**) Top: fluorogenic response in the presence of various interfering agents (from left to right, dopamine, H_2_O_2_, ascorbic acid, NO, nitrate, uric acid). Bottom: response in the presence of day light. 5b. Histogram showing selectivity of NO over other interferences, concentration of interferences is 50 μM & FL spectra recorded in the presence of interferences.

The interference effect was tested with the same concentration of ascorbic acid, dopamine, nitrate, hydrogen peroxide and uric acid. Figure 6a shows fluorescence changes observed during the addition of NO and other interferences (top). Fluorescence quenching occurs more strongly in the presence of NO. The images below show the appearance in daylight. Figure 6b shows the histogram showing the comparison of quenching of NO and various interferences with PEI-CDs and fluorescence spectra recorded in the presence of interferences. The quenching in the presence of NO occurs due to the photoinduced electron transfer from the PEI-CDs to the blue coloured ferrocene-hyponitrite intermediate. The concentration of PEI-CDs is selected from the linear response range (0–0.5 mg/mL) and the concentration Fc_aq_ (1 mM) is chosen that its absorption does not influence the absorption of the PEI-CDs. The interference effect shows that 90% of the quenching is observed by adding 50 μM NO. The detection limit of the sensor system was 500 nM [see Supplementary Figure S8]. Our future work will focus on improving the detection limit by functionalising Fc_aq_ on thePEI-CDs surface and extending this work to neutral pH.

### Triple channel sensing of NO

**Figure 7:**
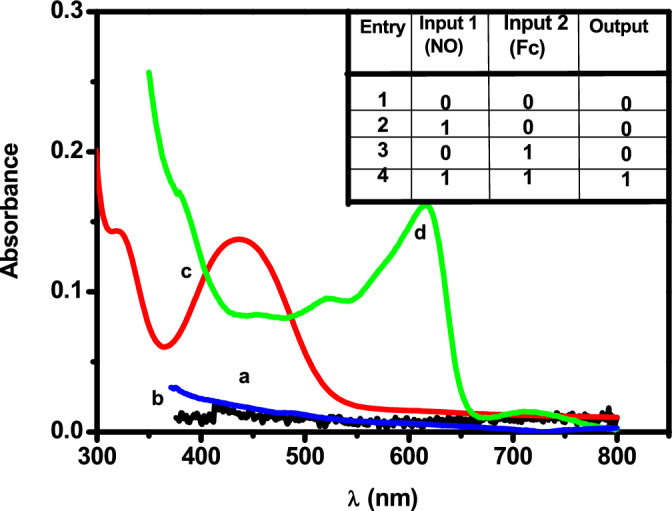
Absorbance spectra (**a**) H_2_SO_4_, (**b**) NO in H_2_SO_4_, (**c**) Fc_aq_ in H_2_SO_4_, (**d**) Fc_aq_ + NO in H_2_SO_4_. Inset shows truth table for AND gate (output is the absorption at 618 nm).

In this work, we reported a triple channel sensor for NO (colorimetric, fluorogenic and electrochemical). To the best of our knowledge, this was one of the first reports of a triple channel probe for NO. Figure 7 shows the absorption spectra recorded for Fc_aq_ and Fc_aq_in the presence of NO. Fc_aq_ shows absorption at 430nm and with the addition of NO absorption at 430 nm decreases and a new peak increase at 618nm due to ferrocene hyponitrite formation. It is important to note that colour change is one of the most convenient methods of classical chemical analysis due to its clarity and inexpensiveness.

Figure 7 describes how absorbance-based detection of NO is similar to the performance of the molecular logic gate “AND”. The absorption of the ferrocenyl cation observed at 618 nm is used as the output signal of the AND gate. NO and Fc_aq_ are considered as the two inputs. In Fig. 7a–c correspond to the inputs (0, 0) (1, 0) and (0, 1), and at 618 nm, no absorption is observed in the electrolyte medium (H_2_SO_4_) in which the reaction takes place as well as in bare NO. Ferrocene shows absorption at 430 nm. The truth table shown in the inset of Fig. 7 represents the various combinations of the two inputs and the respective outputs obtained. Only the presence of the two inputs, Fc and NO can cause the absorption at 618 nm and this process is analogous to the AND gate. Presence of at least one input or absence of both inputs → output = 0 (no absorption observed at 618 nm).

On the other hand, the fluorescence quenching of PEI-CDs at 447 nm by blue ferrocene hyponitrite, as discussed in this article forms the second channel for NO detection. Since quenching (turn off) was only observed upon NO addition, this method proved to be selective. The fluorescence response due to the addition of NO could also be observed with the naked eye when PEI-CDs were illuminated with UV light upon excitation at 360 nm. The concept of NO sensing based on quenching the fluorescence of PEI-CDs is similar to the NAND molecular logic gate, where emission at 420 nm with FL intensity > 23% is considered as the output. The Truth table and fluorescence spectra are shown in Fig. 3b.Presence of at least one input or absence of both inputs → output = 1. But the presence of both inputs, input1 = input2 → output = 0^81^.

**Figure 8:**
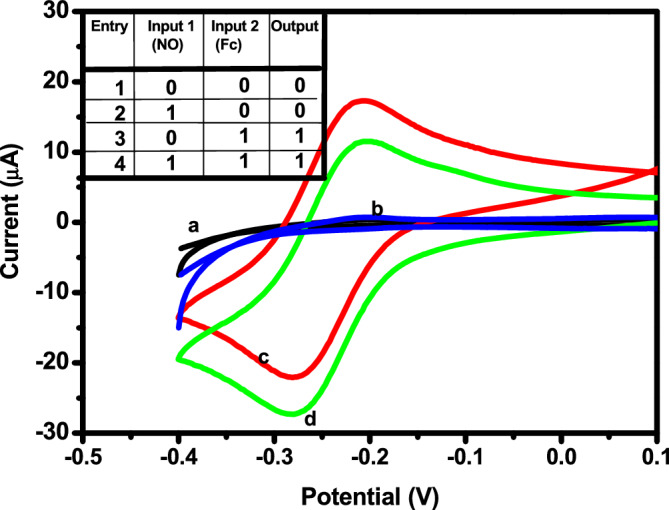
Electrochemical response (**a**) bare gold electrode, (**b**) bare gold electrode in the presence of NO (**c**) Fc_aq_, (**d**) Fc_aq_ + NO—in the medium 0.5 M H_2_SO_4_ vs Hg/Hg_2_SO_4_.

Electrochemical sensing offers potential dependent selectivity, simplicity in detection and is suitable for field-based measurements. We have previously shown that selective electrocatalytic sensing of NO down to nM is possible in the presence of ferrocene^59–61,63^. Figure 8 shows the redox behaviour of Fc_aq_ in aqueous condition (c), which is due to ferrocene–ferrocenyl cation conversion. After the addition of NO the current on the reduction side increased, showing the electrocatalytic response. During the addition of NO, a blue coloured intermediate is formed, which undergoes reduction leading to the regeneration of ferrocene. This happens at a potential of −0.3 V vs Hg/Hg_2_SO_4_. The addition of other interfering substances such as dopamine, H_2_O_2_, ascorbic acid, nitrate, uric acid did not lead to any significant change. The electrochemical sensing of NO is analogous to that of themolecular logic gate ID (Fig. 8)^82^. The voltammetric response of the bare gold electrode in the supporting electrolyte, corresponding to the input (0,0) and of the NO added to the supporting electrolyte, corresponding to the input (1,0) show no redox features in the investigated potential range. Fc_aq_ has a nearly reversible (∆Ep = 83 mV) peak at −0.3 V, corresponding to the input (0, 1). When both inputs (1, 1) are present, there is a current increase at −0.3 V, due to the electrocatalytic reduction of NO. The output is the voltammetric current (> 5μA) at −0.3 V. The absence of both input results in an output = 0. If input 2(ferrocene) = 1 → output = 1, the presence of both inputs give output = 1. Figure S9 (see Supplementary Information) shows a schematic representation of the triple channel sensor for NO in the presence of the common probe Fc_aq_. The comparison of analytical values of various NO sensors is listed in Table 1.

**Table 1:** Comparison of analytical figures of various NO sensors.

No	Probe for NO detection	Mode of detection	Detection limit
1	Griess assay^83,84^	Colorimetric	0.5 μM
2	2,3-diaminonaphthalene^85,86^	Fluorescence	10 nM
3	Diaminofluoresceines^87–89^	Fluorescence	5 nM
4	Copper complex^90^	Fluorescence	10 nM
5	Cobalt complex^91^	Fluorescence	30 nM
6	Haemoglobinimmobilised on pyrolitic graphite surface^92^	Electrochemical reduction	40 nM
7	Nafion/lead rhuthenatepychlore electrode^93^	Electrochemical reduction	4.8 nM
8	3D grapheme^94^	Electrochemical oxidation	16 nM
9	CD’s doped with Nitrogen^95^	Fluorescence	0.3 mM
10	CdSe/ZnS^96^	Fluorescence	3.3 μM
11	Rho-NO^97^	Fluorescence	0.06 μM
12	Perovskite quantum dots^98^	Fluorescence	0–1000 ppm
13	Ferrocene^63,65–67^	Colorimetric Electrochemical	600 nM 50 nM
14	Ferrocene (present work)	Fluorogenic	500 nM

## Conclusions

We have demonstrated a new fluorogenic sensor for NO in aqueous state based on carbon nanodots. Fluorescence detection is based on the quenching of PEI-CDs caused by the blue coloured hyponitrite intermediate between ferrocene and NO. The photoinduced reduction of the blue coloured intermediate by the PEI-CDs is responsible for the quenching phenomenon. The linear response was found to be 1 × 10^–6^ M to 5 × 10^–4^ M. We also demonstrated how a single entity ferrocene simultaneously functions as a triple channel probe for NO detection–colorimetric, voltammetric and fluorogenic with detection limits of 600 nM, 50 nM and 500 nM respectively. It is noteworthy that the triple channel sensing of NO is analogous to the operation of three logic gates. The present work raises concerns in two directions, namely the feasibility of NO detection under neutral detection and the improvement of sensitivity. However, acidic conditions prevail at the tumour site and therefore an acidic environment is suitable for theranostic applications of NO. Further work is underway to introduce sulfonic acid functionalities to maintain acidity at the site of carbon dots, extend the detection of NO to neutral pH, and improve the sensitivity suitable for NO detection at the cellular level.

### Supplementary Information

Supplementary Information.

## Data Availability

All the datas generated during the study are included in the manuscript and in supplementary information. Any other datas clarification needed are available from corresponding author on reasonable request.
