# Supplementary material for: Ferrocene probe-assisted fluorescence quenching of PEI-carbon dots for NO detection and the logic gates based sensing of NO enabled by trimodal detection
Source: Sci Rep. 2024 May 6;14:10402. doi: 10.1038/s41598-024-61117-z (PMC11074144; doi:10.1038/s41598-024-61117-z)
Supplement: Supplementary file 1 — Supplementary Information. [file 41598_2024_61117_MOESM1_ESM.doc]

**Supporting Information**

Ferrocene probe-assisted fluorescence quenching of PEI-carbon dots for NO detection and the logic gates based sensing of NO enabled by trimodal detection

*Priya S. ^a^, Sheela Berchmans* ^b^*

a-NSS college nemmara

b.Electrodics and Electrocatalysis division, Central Electrochemical Research Institute, Karaikudi, Tamilnadu-630006-INDIA

(a)

**(b)**

**Figure S1. Zeta potential analysis of PEI-CD (a) as synthesised (b) at pH=3**

**Figure S2. Linear dependence of PEI-CD from 0.1-0.6mg/ml**

**Figure S3. Emission spectra of CD**

**Figure S4. Excitation at different wavelength for CDs+ Fc_aq_**

**FigureS5- Cyclic voltammograms of bare and PEI-CDs modified GC in 0.5 M H_2_SO_4_**

**Calculation of active surface area of modified and unmodified electrodes**

**I_p_ = -(2.99 X 10^5^) n (α_c_ n_α_)^1/2^ A c^∞^ (D_o_ ʋ)^1/2^**simplified Nicholson shain eq

n_α_ denotes the number of electrons transferred and α_c_is the transfer coefficient. D_o_,c**^∞^**, ʋ are the diffusion coefficient, molar concentration and scan rate terms respectively. In irreversible conditions, the α_c_ n_α_term can be calculated from the equation:


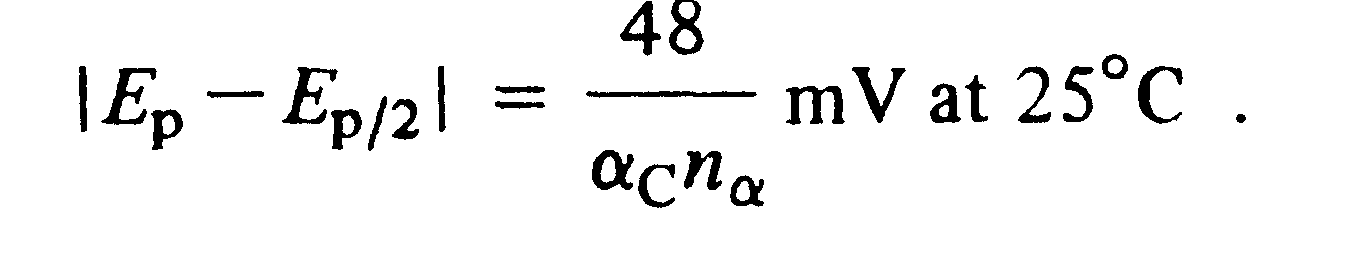


where Ip and ʋ are the peak current (A) and scan rates (V/s) respectively. The constant terms which includes ‘c’ which is the molar concentration of the redox active species (mol/cm^3^), ‘n’ is the number of electrons involved in the redox cycle (n=1 in case of ferrocyanide/ferricyanide system) and D as the diffusion coefficient (cm^2^/s). Ep/2 is the potential at ½ of Ip


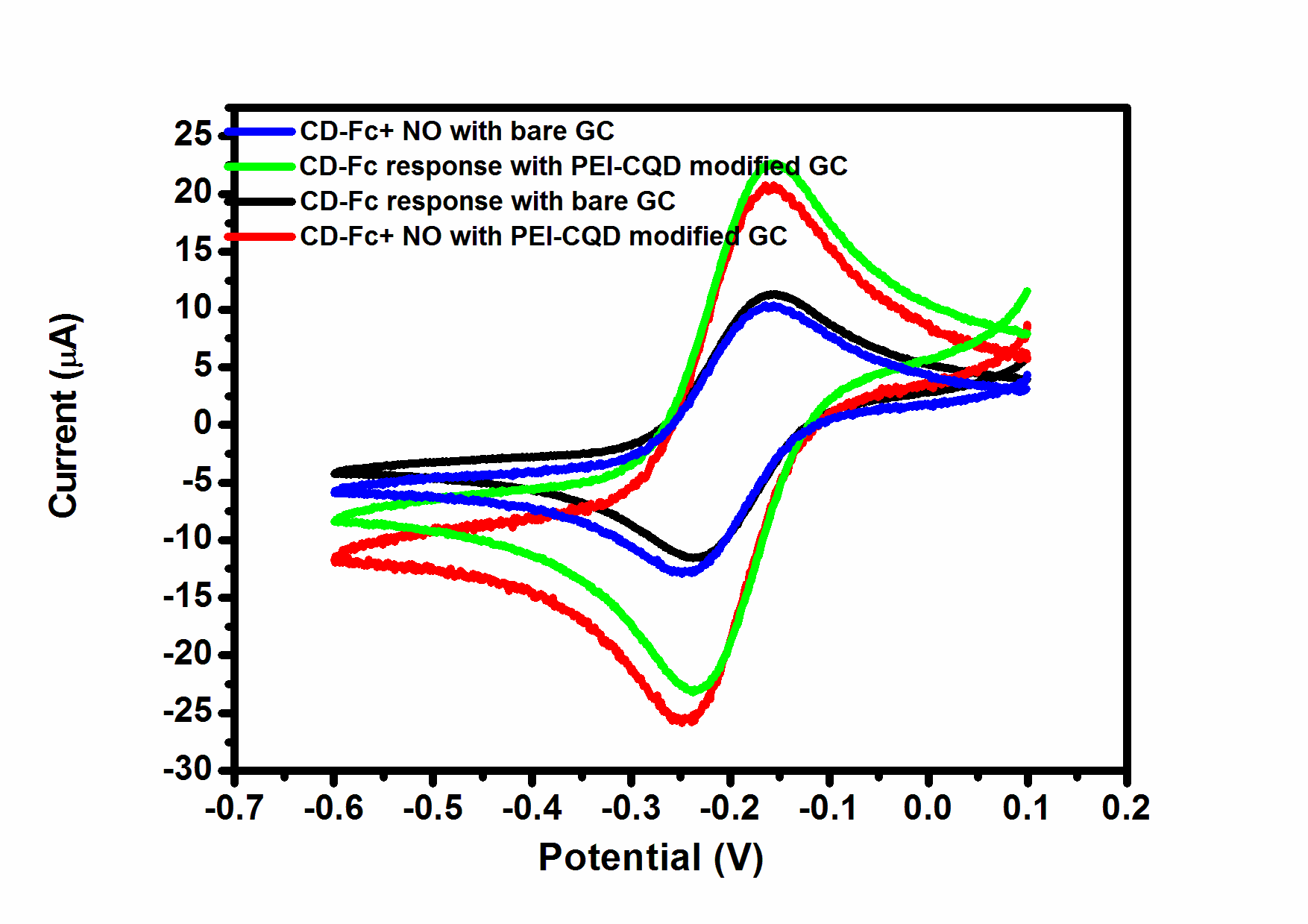


a

b

c

d

**Figure S6. Cyclic voltammetric response of** Fc_aq_ **(a - bare GCE, c-PEI-CD modified GCE),** Fc_aq_ **in the presence of NO (b-bare GCE, d - PEI-CDs modified GCE)**


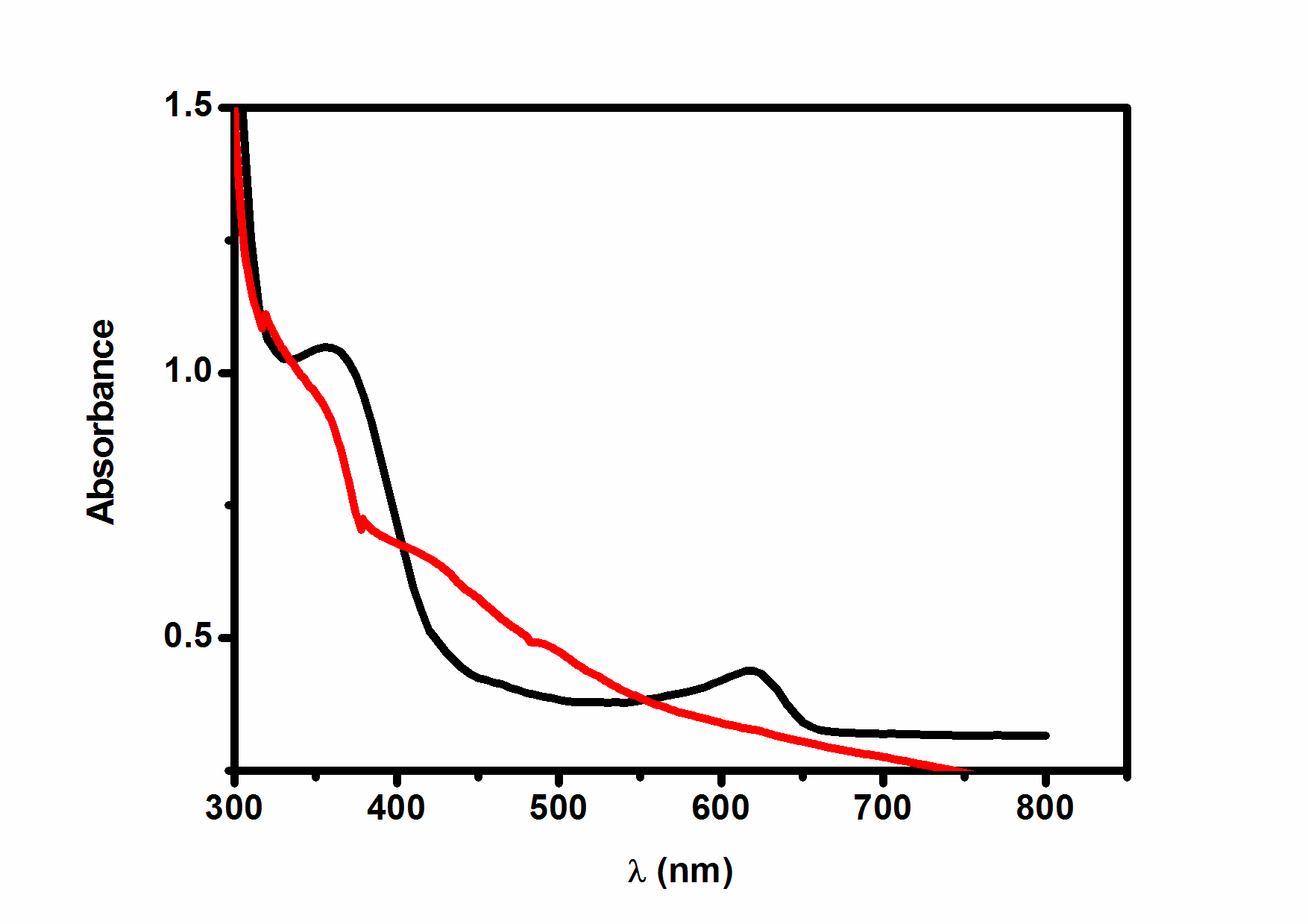


**Figure S7 – UV-Vis spectra reccorded before (black) and after (red) photoexcitation of PEI-CDs in the presence of intermediate.**

**Figure S 8. Calibration plot for the FL response of PEI- CD in the presence of different conc. of NO. Inset shows FL spectra**


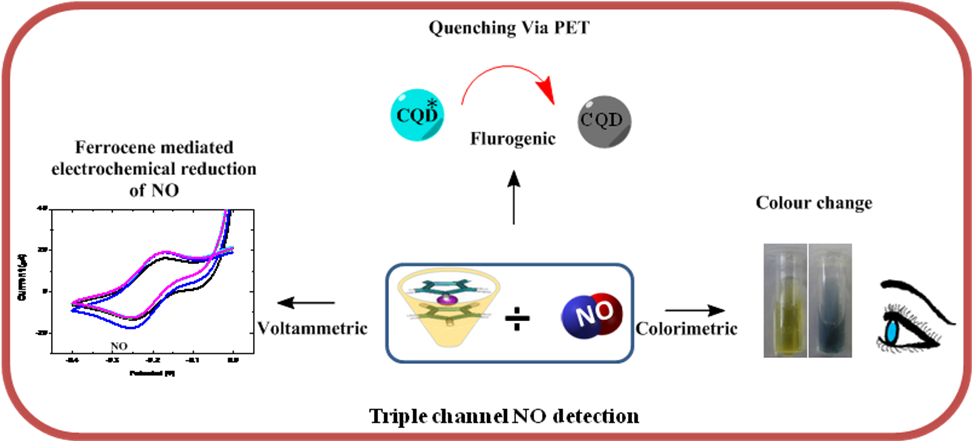


**Figure S 9- Schematic representation of triple channel sensing**
